# Half-quantized helical hinge currents in axion insulators

**DOI:** 10.1093/nsr/nwad025

**Published:** 2023-02-06

**Authors:** Ming Gong, Haiwen Liu, Hua Jiang, Chui-Zhen Chen, X-C Xie

**Affiliations:** International Center for Quantum Materials, School of Physics, Peking University, Beijing 100871, China; Center for Advanced Quantum Studies, Department of Physics, Beijing Normal University, Beijing 100875, China; School of Physical Science and Technology, Soochow University, Suzhou 215006, China; Institute for Advanced Study, Soochow University, Suzhou 215006, China; School of Physical Science and Technology, Soochow University, Suzhou 215006, China; Institute for Advanced Study, Soochow University, Suzhou 215006, China; International Center for Quantum Materials, School of Physics, Peking University, Beijing 100871, China; CAS Center for Excellence in Topological Quantum Computation, University of Chinese Academy of Sciences, Beijing 100190, China

**Keywords:** axion insulator, parity anomaly, half-quantization, Goos-Hänchen effect, topological magnetoelectric effect

## Abstract

Fractional quantization can emerge in noncorrelated systems due to the parity anomaly, while its condensed matter realization is a challenging problem. We propose that in axion insulators (AIs), parity anomaly manifests a unique fractional boundary excitation: the half-quantized helical hinge currents. These helical hinge currents microscopically originate from the lateral Goos-Hänchen (GH) shift of massless side-surface Dirac electrons that are totally reflected from the hinges. Meanwhile, due to the presence of the massive top and bottom surfaces of the AI, the helical current induced by the GH shift is half-quantized. The semiclassical wave packet analysis uncovers that the hinge current has a topological origin and its half quantization is robust to parameter variations. Lastly, we propose an experimentally feasible six-terminal device to identify the half-quantized hinge channels by measuring the nonreciprocal conductances. Our results advance the realization of the half-quantization and topological magnetoelectric responses in AIs.

## Teaser

Half-quantized helical hinge current, which originates from the topological literal shift of electrons on the metallic side surface, serves as the fingerprint signature of the axion insulator.

## INTRODUCTION

Fractional quantization in condensed matter materials is usually accompanied by the emergence of quasi-particles driven by strong correlations. A prominent example is the fractional quantum Hall effect [1–3], which is the fairyland of fractionally charged quasi-particle excitations, and has attracted intense attention in the condensed matter community since its discovery. Interestingly, fractional quantization can also emerge in noncorrelated systems. Such a fractional quantization is triggered by parity anomaly [4–6], which generates a parity-violating current with the half-integer-quantized Hall conductance. However, realizing the parity anomaly in (2+1) dimensions and observing the half-quantized transport signals in condensed matter systems have been challenging problems for over 40 years [7–11]. A critical issue is that the bulk-boundary correspondence principle implies that some kind of half-quantized boundary excitations should exist in the parity anomaly systems. Unfortunately, the physical picture of such excitations, their material realization and the roadmap of their experimental characterization are elusive.

Encouragingly, the axion insulator (AI) [12–15] provides an ideal platform to realize parity anomaly on its top and bottom surfaces [16–18]. As a nontrivial topological phase, the AI manifests a unique topological magnetoelectric (TME) effect [15,19–24] and has stimulated extensive research interests [25–48]. Experimentally, the zero Hall conductance plateau has been observed in magnetic topological insulator (TI) heterostructures [28–30] and the antiferromagnetic TI $\rm MnBi_{2}Te_{4}$ [33]. However, the zero Hall plateau cannot provide smoking-gun evidence of the AI, and, more importantly, parity-anomaly-induced half-quantized signals in AIs have not been observed. Such a dilemma originates from shallow knowledge of the boundary excitations in AIs. To give a definitive answer, a concrete and in-depth understanding of the parity-anomaly-induced boundary excitations in the AI is highly desirable.

**Figure 1.**
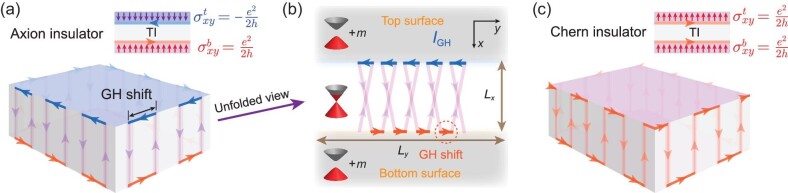
Schematics of the half-quantized hinge currents. Half-quantized hinge currents originate from the GH shift currents of side-surface electrons that bounce back and forth between gapped top and bottom surfaces in the AI (a) and the CI (c). (b) Unfolded view of the top, side and bottom surfaces of the AI. The trajectory of a massless electron illustrates the formation of the GH shift current *I*_GH_. When the contributions from all the electrons are considered, the trajectory segments on the hinges are merged together, leading to net helical hinge currents.

In this work, we find that parity anomaly in AIs induces a unique boundary excitation: the half-quantized helical hinge currents. Based on the semiclassical wave packet dynamics, we establish the microscopic picture of these hinge currents. We proposed that the massless Dirac electrons on the side surfaces of the AI undergo a lateral Goos-Hänchen (GH) shift when they are reflected from the massive top or bottom surface (see Fig. 1), reminiscent of the GH shift [49–53] of the totally reflected light beam. Moreover, due to the breaking of time-reversal ($\mathcal {T}$) symmetry, the GH shift favors a specific direction on the hinge. Consequently, helical GH shift currents accumulate on the hinges of the AI when side-surface electrons bounce back and forth between the gapped top and bottom surfaces (see Fig. 1(a) and (b)), which is distinct from the chiral net currents on the edge of the Chern insulator (CI) (see Fig. 1(c)). Interestingly, we find that the differential GH shift current δ*I*_GH_ is exactly half-quantized with respect to the differential Fermi energy δ*E*_F_, i.e. δ*I*_GH_ = *e*δ*E*_F_/2*h*. We demonstrate that the GH shift current originates from the nonvanishing Berry curvature during the scattering process, and its half-quantization is robust to the variation of parameters. Then, we numerically verify the half-quantized helical hinge currents through a three-dimensional (3D) lattice model. Finally, we propose a six-terminal device to identify the half-quantized hinge currents in the AI by measuring the nonreciprocal conductances.

## MODEL HAMILTONIAN AND THE GOOS-HÄNCHEN SHIFT

To begin with, we model the AI by a 3D magnetic TI, of which the top and bottom surfaces are gapped oppositely while the side surfaces remain gapless. Such a model is in accordance with the experimental setups utilized to realize the AI state in magnetic TI heterostructures or the antiferromagnetic TI $\rm MnBi_{2}Te_{4}$ [28–30,33]. According to the bulk-boundary correspondence theorem, the top/bottom surface and the side surface of the AI can be described by the 2D effective Hamiltonians


(1) \begin{eqnarray*} \mathcal {H}=\left\lbrace \begin{array}{@{}l@{\ }l@{}}\hbar v_{\rm F}(-i\sigma _{x}\partial _{x}-i\sigma _{y}\partial _{y})-U&({\rm side}),\\ \hbar v_{\rm F}(-i\sigma _{x}\partial _{x}-i\sigma _{y}\partial _{y}) +\, m\sigma _{z}\\ \quad&({\rm top/bottom}). \end{array}\right.\\ \end{eqnarray*}

Here, *v*_F_ denotes the Fermi velocity, *U* is the gate potential and *m* is the mass term induced by the magnetization. In the following discussions, we unfold the top, side and bottom surfaces of the AI (see Fig. 1(a)) into the *x*-*y* plane (see Fig. 1(b)).

**Figure 2.**
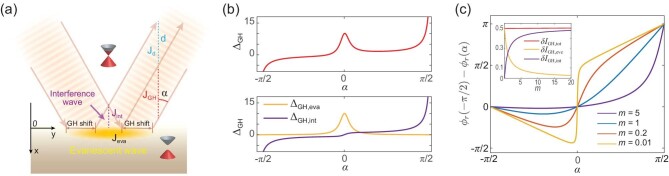
Microscopic mechanism of the hinge currents due to the GH shift. (a) Sketch of the scattering process on the hinge, where a beam of massless Dirac electrons is totally reflected from a massive barrier. (b) The GH shift versus α with *U* = 1, *m* = 0.1 and *E* = 0. The upper panel shows the total GH shift Δ_GH_ and the lower panel shows its contributions from the evanescent wave (Δ_GH,eva_) and the interference wave (Δ_GH,int_). (c) Reflection phase difference versus α for different *m*. The inset shows the evanescent wave and interference wave contributions to δ*I*_GH_ (in units of *e*δ*E*_F_/*h*) versus *m*.

We use the probability flux method (see [51,52] and the online supplementary material) to calculate the GH shift on the hinge of the AI. As shown in Fig. 2(a), we place the massless side surface in *x* ≤ 0 and the massive top/bottom surface in *x* > 0. Firstly, we solve the scattering problem by matching $\psi _{\rm {int}}( {\boldsymbol r})=\psi _{\rm {in}}+r\psi _{\rm {re}}$ (*x* ≤ 0) and $\psi _{{\rm eva}}(\boldsymbol {r})=e^{-\kappa x+ik_{y}y}\psi (0,0)$ (*x* > 0) at *x* = 0, where $\psi _{\rm {int}}(\boldsymbol {r})$ is the interference superposition of the incident plane wave $\psi _{\rm {in}}(\boldsymbol {r})=e^{ik_{x}x+ik_{y}y}[e^{-i{\alpha }/{2}}, e^{i{\alpha }/{2}}]^{T}/\sqrt{2}$ and reflected plane wave $\psi _{\rm {re}}(\boldsymbol {r})=e^{-ik_{x}x+ik_{y}y}[e^{-i{(\pi -\alpha )}/{2}}, e^{i{(\pi -\alpha )}/{2}}]^{T}/\sqrt{2}$ in the massless region. Here $\psi _{{\rm eva}}(\boldsymbol {r})$ is the evanescent wave in the massive region, α = arctan(*k_y_*/*k_x_*) denotes the incident angle (see Fig. 2(a)) and $\boldsymbol {r}=(x,y)$. Here we only consider the total reflection process, which captures the physics within the magnetization gap. The reflection coefficient $r=e^{i\phi _{\rm r}}$ is given in the online supplementary material. Then, imagine that the incident and reflected waves have finite width, as depicted in Fig. 2(a); the GH shift can be obtained under the probability flux conservation constraint. As labeled in Fig. 2(a), *J*_int_, *J*_eva_ and *J*_GH_ represent the fluxes carried by the interference wave, the evanescent wave and the part proportional to the GH shift. We denote by *J*_d_ the flux through the cross section colored blue with width *d*. Suppose that the probability density of the incident/reflected beam is normalized as $\psi _{\rm in}^{\dagger }({\boldsymbol r})\psi _{\rm in}({\boldsymbol r})=\psi _{\rm re}^{\dagger }({\boldsymbol r})\psi _{\rm re}({\boldsymbol r})=1$; then


(2a) \begin{eqnarray*} J_{\rm {d}}=v_{\rm F}d{\rm sin}\alpha , \end{eqnarray*}

(2b) \begin{eqnarray*} J_{\rm {GH}}=v_{\rm F}\Delta _{\rm GH}\rm {cos}\alpha , \end{eqnarray*}

(2c) \begin{eqnarray*} J_{\rm {eva}}=v_{\rm F}\frac{{\rm sin}\alpha +{\rm cos}\phi _{\rm r}}{\kappa }, \end{eqnarray*}

(2d) \begin{eqnarray*} J_{\rm {int}}(d)=v_{\rm F}\bigg [d{\rm sin}\alpha +\frac{{\rm sin}(\phi _{\rm r}+k_{x}d)}{k_{x}}-\frac{{\rm sin}\phi _{\rm r}}{k_{x}}\bigg ]. \end{eqnarray*}

The flux conservation condition imposes that *J*_int_ + *J*_eva_ = *J*_GH_ + *J*_d_. The GH shift as a function of *d* reads


(3) \begin{eqnarray*} \Delta _{\rm GH}(d)&=&\frac{{\rm sin}\alpha +{\rm cos}\phi _{\rm r}}{\kappa {\rm cos}\alpha }-\frac{{\rm sin}\phi _{\rm r}}{k_{x}{\rm cos}\alpha }\\ &&+\, \frac{{\rm sin}(\phi _{\rm r}+k_{x}d)}{k_{x}{\rm cos}\alpha }. \end{eqnarray*}

In the semiclassical limit, the *d* dependence of Δ_GH_(*d*) can be averaged out, resulting in the net GH shift


(4) \begin{eqnarray*} \Delta _{\rm GH}=\langle \Delta _{\rm GH}(d)\rangle _{d}=\frac{{\rm sin}\alpha +{\rm cos}\phi _{\rm r}}{\kappa {\rm cos}\alpha }-\frac{{\rm sin}\phi _{\rm r}}{k_{x}{\rm cos}\alpha }. \end{eqnarray*}

In the upper panel of Fig. 2(b), we plot Δ_GH_ as a function of the incident angle α with fixed *E, U* and *m*. For glancing incidence, i.e. α → ±π/2, Δ_GH_ diverges but with opposite sign for α = π/2 and α = −π/2. Such behavior indicates that at large incident angles the GH shift has the same direction as the incident wave, and thus does not show the chiral feature. However, Δ_GH_ peaks for vertical incidence, i.e. α → 0, clearly showing that the GH shift favors a specific direction and manifests the chiral feature. The chiral Δ_GH_ further implies that the net GH shift current accumulated on the hinge is chiral. We then decompose Δ_GH_ according to its contributions from the evanescent wave Δ_GH,eva_ and the interference wave Δ_GH,int_. As will be discussed later, Δ_GH,eva_ and Δ_GH,int_ introduce the components of the shift current with different decay laws. The lower panel of Fig. 2(b) shows that the chiral part of Δ_GH_ is mostly carried by Δ_GH,eva_, which lies on the $\mathcal {T}$-symmetry-broken top/bottom surface of the AI. In contrast, the nonchiral part, especially for glancing incidence α → ±π/2, is mostly carried by Δ_GH,int_.

## HALF-QUANTIZED SHIFT CURRENT

To give a microscopic picture of the chiral hinge current induced by the GH shift, we consider the electron in the scattering problem as a point particle, which bounces back and forth between double massive barriers as sketched in Fig. 1(b). Suppose that the width between the opposite hinges is *L_x_*; thus, the average time interval between two consecutive bounces is $\Delta \tau =2L_{x}/v_{\rm F}\rm {cos}\alpha$ for −π/2 < α < π/2. When it bounces off the hinge, it undergoes a lateral GH shift Δ_GH_. Such a lateral shift induces an anomalous velocity of electrons along the hinge as


(5) \begin{eqnarray*} v_{\rm GH}=\frac{\Delta _{\rm GH}}{\Delta \tau }=\frac{\Delta _{\rm GH}v_{\rm F}{\rm cos}\alpha }{2L_{x}}. \end{eqnarray*}

The total GH shift current induced by *v*_GH_ is obtained by counting the contributions of all filled electrons as


(6) \begin{eqnarray*} I_{\rm GH}=\sum _{\rm filled} \frac{ev_{\rm GH}}{L_{y}}=\frac{e}{h}\int _{-\infty }^{E_{\rm F}}dE\int _{-K}^{K}\frac{dk_{y}}{2\pi }\Delta _{\rm GH}, \end{eqnarray*}

where *L_y_* is the circumference of the side surface and *K* = (*E* + *U*)/ℏ*v*_F_ is the Fermi wave vector at energy *E*. Details of the calculations can be found in the online supplementary material. By employing the stationary phase method (see [50,54–56] and the online supplementary material), we find that the GH shift can be written as Δ_GH_ = −∂φ_r_/∂*k_y_*. Substituting this result into (6), we obtain the differential shift current δ*I*_GH_ = δ*E*_F_[φ_r_(−π/2) − φ_r_(π/2)]*e*/2π*h*.

In Fig. 2(c), we plot φ_r_ as a function of α for different *m*. It is clear that φ_r_(−π/2) − φ_r_(π/2) = π and robust to the variation of *m* (see the online supplementary material). Therefore, δ*I*_GH_ is exactly half-quantized with respect to δ*E*_F_ as


(7) \begin{eqnarray*} \delta I_{\rm GH}=\frac{e}{2h}\delta E_{\rm F}. \end{eqnarray*}

Moreover, δ*I*_GH_ can be decomposed according to its contributions from the evanescent wave and the interference wave, i.e. δ*I*_GH_ = δ*I*_GH, eva_ + δ*I*_GH,int_. We emphasize that δ*I*_GH,int_ shows a power-law (*x*^−1/2^) decay from the hinge, while δ*I*_GH,eva_ decays exponentially from the hinge (*e*^−*x*/λ^); see the online supplementary material. Therefore, δ*I*_GH_ is different from the current carried by the topologically protected edge or hinge state, which decays exponentially on both sides of the hinge. The inset of Fig. 2(c) plots δ*I*_GH,eva_ and δ*I*_GH,int_ versus *m*. The differential shift current δ*I*_GH_ is mainly contributed from δ*I*_GH,eva_ when *m* is small. As *m* increases, the contribution from δ*I*_GH,int_ becomes dominant.

## TOPOLOGICAL ORIGIN OF THE HALF QUANTIZATION

**Figure 3.**
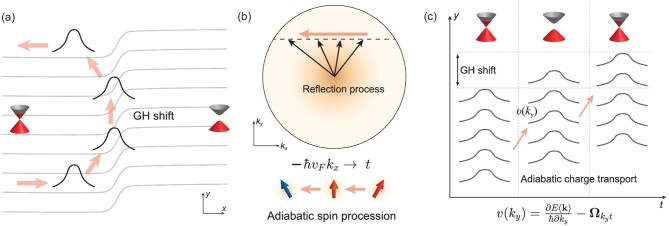
Topological origin of the half-quantized GH shift current. (a) Sketch of the process where a wave packet of a massless Dirac electron bounces off a massive barrier, undergoing a lateral GH shift in the *y* direction. (b) Sketch of the reflection process of the wave packet in the momentum space where *k_y_* is unchanged due to the translation symmetry in the *y* direction. During the reflection the energy *E* is conserved; therefore, *k_x_* and the mass *m* of the local Hamiltonian are varying under the constraint $\hbar ^{2}v_{\rm F}^{2}k_{x}^{2}+\hbar ^{2}v_{\rm F}^{2}k_{y}^{2}+m^{2}=E^{2}$. Such a reflection process can also be understood as the 1D charge transport problem in the *y* direction when we take *t* = −ℏ*v*_F_*k_x_* as the virtual time. The effective time-dependent Hamiltonian can be viewed as a Zeeman type $H(t)={\boldsymbol B}(t)\cdot {\boldsymbol \sigma }$ with ${\boldsymbol B}(t)=[-t,\hbar v_{\rm F}k_{y},m(t)]$ and the reflection process is simplified to an adiabatic spin procession for fixed *k_y_* and *E*. (c) Sketch of the adiabatic charge transport in the *y* direction. The nontrivial Berry curvature $\Omega _{k_{y}t}$ induces an anomalous velocity *v*(*k_y_*) in the *y* direction. After the reflection, the total contribution from the adiabatic current gives rise to the GH shift.

We provide a topological viewpoint of the half-quantized GH shift current in the frame of adiabatic charge transport theory [57–60]. Here, we use Hamiltonian $\mathcal {H}(\boldsymbol {r})=\hbar v_{\rm F}(-i\sigma _{x}\partial _{x}-i\sigma _{y}\partial _{y})+m(x)\sigma _{z}$ to describe the scattering process, where *m*(*x*) is a smooth function connecting the gapless and the gapped regions with *m*(*x*) → *m* for *x* ≫ 0 and *m*(*x*) → 0 for *x* ≪ 0 (see Fig. 3(a)). During the reflection process, the energy *E* and momentum *k_y_* are conserved. Therefore, the relation $\hbar ^{2}v_{\rm F}^{2}k_{x}^{2}+\hbar ^{2}v_{\rm F}^{2}k_{y}^{2}+m^{2}=E^{2}$ holds. We take *t* = −ℏ*v*_F_*k_x_* as the virtual time and the scattering process is now described by the time-dependent 1D Hamiltonian *H*(*k_y_, t*) = −*t*σ_*x*_ + ℏ*v*_F_*k_y_*σ_*y*_ + *m*(*t*)σ_*z*_, which describes the Zeeman coupling of a Pauli spinor to a time-dependent magnetic field ${\boldsymbol B}(t)=[-t,\hbar v_{\rm F}k_{y},m(t)]$ (see Fig. 3(b)). The study of the GH shift is reduced to the study of an adiabatic charge transport problem in the *y* direction, with an internal adiabatic spin procession under the magnetic field ${\boldsymbol B}(t)$ [61].

The instantaneous eigenstates of *H*(*k_y_, t*) are $|u_{\pm }(k_{y},t)\rangle =[E\pm m,-t+i\hbar v_{\rm F} k_{y}]^{T}/\sqrt{2E(E\pm m)}$, where ‘±’ denotes the spin-up and spin-down components of the spinor. Following the analysis in [60], up to first order in the rate of change in the Hamiltonian, the wave function is given by


(8) \begin{eqnarray*} &&\!\!\!|u_{\pm }(k_{y},t)\rangle\\ &&\!\! -\, i\hbar \sum _{n^{\prime }\ne n}\frac{|u_{n^{\prime }}(k_{y},t)\rangle \langle u_{n^{\prime }}(k_{y},t)|\partial u_{n}(k_{y},t)/\partial t\rangle }{E_{n}-E_{n^{\prime }}}, \\ \end{eqnarray*}

where *n*(*n*′) = ± represents the spin-up or spin-down components. The average velocity for a given *k_y_* is, to first order,


(9) \begin{eqnarray*} v_{n}(k_{y}) &=&\partial E_{n}(k_{y})/\hbar \partial k_{y}\\ && -\, i\bigg [ \bigg \langle \frac{\partial u_{n}}{\partial k_{y}}\bigg | \frac{\partial u_{n}}{\partial t} \bigg \rangle - \bigg \langle \frac{\partial u_{n}}{\partial t}\bigg | \frac{\partial u_{n}}{\partial k_{y}} \bigg \rangle \bigg ] \\ &=&\partial E_{n}(k_{y})/\hbar \partial k_{y}-\Omega ^{n}_{k_{y}t}, \end{eqnarray*}

where $\Omega ^{n}_{k_{y}t}=-2{\rm Im} \langle \partial u^{n}/\partial k_{y}|\partial u^{n}/\partial t \rangle$ is the Berry curvature in *k_y_*-*t* space. We only consider the conduction band with *n* = + where the scattering process happens; thus, from now on we omit the band index *n*. The GH shift (see Fig. 3(c)) in the *y* direction contributed from a given *k_y_* is


(10) \begin{eqnarray*} \Delta _{\rm GH}(k_{y})&=&\int _{-T(k_{y})}^{T(k_{y})}v(k_{y}) dt\\ &=& \int _{-T}^{T}\bigg [ \frac{\partial E(k_{y})}{\hbar \partial k_{y}}-\Omega _{k_{y}t} \bigg ]dt \end{eqnarray*}

with $T(k_{y})=\sqrt{E^{2}-\hbar ^{2}v_{\rm F}^{2}k_{y}^{2}}$. Combining this with (6), one obtains


(11) \begin{eqnarray*} \frac{\delta I_{\rm GH}}{\delta E_{\rm F}}&=&-\frac{e}{h}\int _{-{E}/{\hbar v_{\rm F}}}^{{E}/{\hbar v_{\rm F}}} \frac{dk_{y}}{2\pi } \int _{-T}^{T}\Omega _{k_{y}t} dt\\ &=&-\frac{e}{2\pi h}\Gamma (C), \end{eqnarray*}

where Γ(*C*) is the Berry phase along boundary *C* of the integration manifold. The integration of ∂*E*(*k_y_*)/ℏ∂*k_y_* vanishes because the band structure is symmetric with respect to *k_y_*.

Intuitively, Γ(*C*) = ±π due to the gapless Dirac electrons on the side surfaces of the AI. To pin down the sign of Γ(*C*), and hence the direction of the differential GH shift current, we perform the integral in polar coordinates. Define $k=\sqrt{t^{2}+\hbar ^{2}v_{\rm F}^{2}k_{y}^{2}}$, *t* = *k*cosθ and ℏ*v*_F_*k_y_* = *k*sinθ. The Berry curvature under the polar coordinate system becomes $\Omega _{k\theta }=i[\langle \partial u/\partial k| \partial u/\partial \theta \rangle - \langle \partial u/\partial \theta | \partial u/\partial k \rangle ]/k= -{\rm sgn}(m)/2E\sqrt{E^{2}-k^{2}}$ with $|u(k,\theta )\rangle =[E+m,-ike^{-i\theta }]^{T}/\sqrt{2E(E+ m)}$. The Berry phase


(12) \begin{eqnarray*} \Gamma (C)&=&\int _{0}^{2\pi } d\theta \int _{0}^{E} kdk \frac{-{\rm sgn}(m)}{2E\sqrt{E^{2}-k^{2}}}\\ &=&-{\rm sgn}(m)\pi . \end{eqnarray*}

Therefore, we conclude that the half-quantized chiral GH shift current δ*I*_GH_/δ*E*_F_ = sgn(*m*)*e*/2*h* is protected by the π Berry phase of the massless Dirac electron while its direction is determined by the mass *m* of the massive barrier.

## VISUALIZING THE HALF-QUANTIZED HINGE CURRENT DISTRIBUTION

The half-quantized hinge current can be numerically visualized based on the 3D magnetic TI Hamiltonian. The model Hamiltonian is given by *H* = *H*_0_ + *H*_M_, where *H*_0_ = ∑_*i*=*x, y, z*_*Ak_i_*τ_*x*_ ⊗ σ_*i*_ + (*M*_0_ − *Bk*^2^)τ_*z*_ ⊗ σ_0_ is the nonmagnetic part and $H_{\rm M}=M(\boldsymbol {r})\tau _{0}\otimes \sigma _{z}$ represents the magnetization term [62,63]. Here, σ_*i*_ and τ_*i*_ are the Pauli matrices acting on the spin and orbital spaces, respectively. The current density [16] in the *y* direction across the *x*-*z* plane with $\boldsymbol {r}=(x,z)$ is


(13) \begin{eqnarray*} &&J_{y}(E,\boldsymbol {r})\\ && =-\frac{e}{\pi h}\int _{-\pi }^{\pi }{\rm ImTr}\bigg [\frac{\partial H(k_{y})}{\partial k_{y}}G^{r}_{k_{y}}(E,\boldsymbol {r},\boldsymbol {r})\bigg ]dk_{y}, \\ \end{eqnarray*}

where $G^{r}_{k_{y}}(E,\boldsymbol {r},\boldsymbol {r^{\prime }})$ is the retarded Green’s function for the momentum-sliced Hamiltonian *H*(*k_y_*).

**Figure 4.**
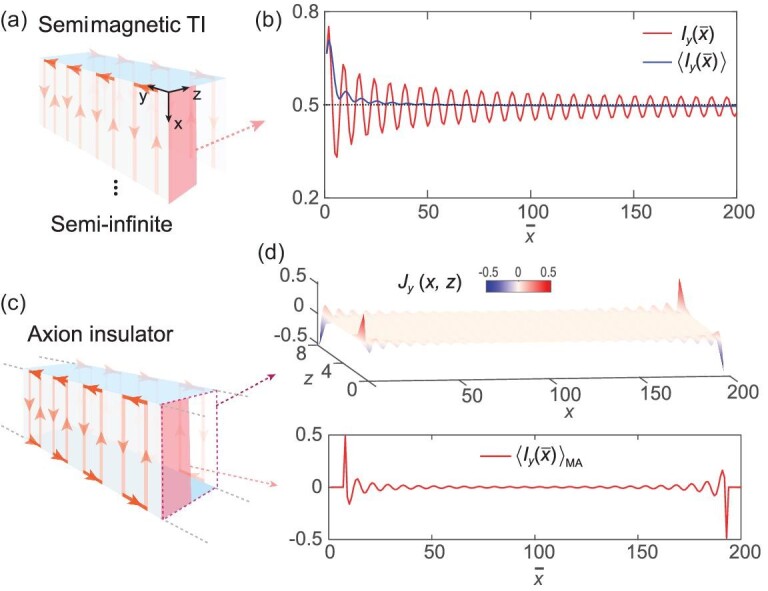
Current density distribution for semimagnetic TI and AI. (a),(c) Schematics of semimagnetic TI and AI (infinite in the *y* direction). The thickness in the *z* direction is *L_z_* = 8. The pink areas in (a) and (c) show the region (0 ≤ *z* ≤ *L_z_*/2) and the dotted box in (c) shows the cross section in the *x*-*z* plane. (b) The current flux through region $[0\leqslant x\leqslant \bar{x},\, 0\leqslant z\leqslant L_{z}/2]$ and $\bar{x}$ averaged $\langle I_{y}(\bar{x}) \rangle$ for semimagnetic TI. (d) The upper panel shows the distribution of *J_y_*(*x, z*) in the *x*-*z* plane. The lower panel shows the moving averaged current flux $\langle I_{y}(\bar{x})\rangle _{\rm MA}$ through the window $[\bar{x}-7\leqslant x\leqslant \bar{x}+7,\, 0\leqslant z\leqslant L_{z}/2]$. Both *J_y_* and *I_y_* are in units of *e*/*h*.

We first evaluate a semimagnetic TI with a gapped top surface in the *y*-*z* plane, which can be viewed as ‘half’ of an AI (see Fig. 4(a)) [39,40,64]. As shown in Fig. 4(b), the current flux $I_{y}(\bar{x})=\int _{0}^{L_{z}/2}dz\int _{0}^{\bar{x}}dx J_{y}(x,z)$ oscillates around 0.5. The $\bar{x}$ averaged $\langle I_{y}(\bar{x}) \rangle =\int _{0}^{\bar{x}}I_{y}(\bar{x}^{\prime })d\bar{x}^{\prime }/\bar{x} \rightarrow 0.5$ clearly shows the half-quantization of the chiral hinge current, which coincides with the GH shift analysis. For an AI where the top and bottom surfaces are gapped with opposite magnetization (see Fig. 4(c) and (d)), a pair of counterpropagating hinge currents emerges on the hinges. The moving averaged current flux $\langle I_{y}(\bar{x})\rangle _{\rm MA}=\int _{0}^{L_{z}/2}dz\int _{\bar{x}-7}^{\bar{x}+7}dxJ_{y}(x,z)$ further demonstrates that the hinge currents are nearly half-quantized and helical.

## EXPERIMENTAL CHARACTERIZATION OF THE HALF-QUANTIZED HELICAL HINGE CURRENTS

**Figure 5.**
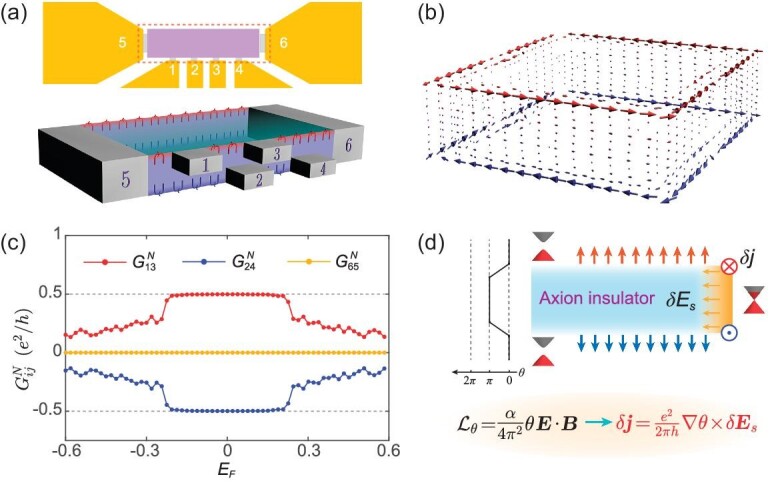
Experimental characterization of the half-quantized helical hinge currents by the nonreciprocal conductance. (a) Schematic of the six-terminal device. Leads 5 and 6 contact to the ends of the AI film. Terminals 1–4 are surface leads with 1 and 3 (2 and 4) contacted near the top (bottom) surface. (b) Local current distribution of the AI. (c) Nonreciprocal conductance $G^{N}_{ij}$ between lead *i* and lead *j* as a function of *E_F_*. (d) Schematic illustration of the relationship between the quantized TME response and the half-quantized helical hinge currents. Here $\delta \boldsymbol {E}_{s}$ is the interface electric field and $\delta \boldsymbol {j}$ is the hinge current.

The half-quantized hinge current δ*I*_GH_ can be experimentally detected by a six-terminal device, as illustrated in Fig. 5(a). Leads 1 and 3 (2 and 4) are contacted near the top (bottom) surface of the AI, while leads 5 and 6 are contacted to the ends of the sample. According to the Landauer-Büttiker formula, the transmission coefficient between terminals *i* and *j* is *T_ij_* = Tr[**Γ**_*i*_**G**^*r*^**Γ**_*j*_**G**^*a*^] and the related differential conductance is *G_ij_* = *e*^2^/*h* · *T_ij_* [65–68]. The measurement method of *G_ij_* is given in the online supplementary material. Here **Γ**_*i*_ is the linewidth function of lead *i* and **G**^*r*(*a*)^ is the retarded (advanced) Green’s function of the AI sample.

To demonstrate the existence of the helical hinge channels, we calculate the nonreciprocal conductance $G^{N}_{ij}=G_{ij}-G_{ji}$ between lead *i* and lead *j*. As shown in Fig. 5(c), in the AI state $G^{N}_{13}=e^{2}/2h$ and $G^{N}_{24}=-e^{2}/2h$ are half-quantized with opposite signs and $G^{N}_{65}=0$. This implies that there exist two counterpropagating half-quantized hinge channels. Moreover, the spatial distribution of the local current *J*_**i** → **j**_(*E*) from site **i** to **j** [69] further reveals the helical signature of the transport hinge currents in the AI, as shown in Fig. 5(b). Since the nonreciprocal conductance $G^{N}_{ij}$ counts the asymmetric part of *J*_**i** → **j**_(*E*), we can deduce that the half-quantized conducting channel originates from the half-quantized chiral GH shift current δ*I*_GH_ on the hinge of the AI. The results are well consistent with those determined from the GH shift (see Fig. 2) as well as the current density distribution (see Fig. 4). Therefore, these transport signals strongly confirm the reliability of the microscopic picture of half-quantized hinge currents proposed in Fig. 1. Since the half-quantized helical hinge currents serve as a fingerprint of the AI, our proposals also promote the experimental identification of AIs.

## DISCUSSION

The half-quantized helical hinge currents can be understood as a consequence of the quantized TME response in the AI [15]. As sketched in Fig. 5(d), a shift of the Fermi energy on the surfaces of the AI induces an interface electric field $\delta \boldsymbol {E}_{\rm s}=-\nabla \delta E_{\rm F}/e$. According to Maxwell’s equations with the axion term, $\delta \boldsymbol {j}=e^{2}/2\pi h\cdot \nabla \theta \times \delta \boldsymbol {E}_{\rm s}$ [15,70] with the spatially varying axion angle θ. The half-quantized hinge current δ*I* = ±*e*δ*E*_F_/2*h* is obtained by integrating $\delta \boldsymbol {j}$ over the corner.

In conclusion, we found that the half-quantized helical hinge currents exist in AIs, and established their microscopic picture based on GH shift currents. The half-quantization of the GH shift current has a topological origin and is robust to the variation of parameters. We numerically demonstrated that the half-quantized hinge channel is reflected by the half-quantized nonreciprocal conductances. Our studies deepen the perception of the boundary excitations of the AI and shed light on the detection of AIs through transport experiments.

## METHODS

### Derivation of the cross-section current density

In calculating the cross-section current density, we take the system to be infinite in the *y* direction. The cross section in the *x*-*z* plane is finite in the *x* and *y* directions for the AI and the CI, and semi-infinite in the *x* direction for the semimagnetic TI. Since the Hamiltonian is infinite in the *y* direction for the three cases, *k_y_* is a good quantum number and the total Hamiltonian *H* can be decomposed into summations of the momentum-sliced Hamiltonians as


(14) \begin{eqnarray*} H=\int _{-\pi }^{\pi }\frac{dk_{y}}{2\pi }H(k_{y},\boldsymbol {r}), \end{eqnarray*}

where $H(k_{y},\boldsymbol {r})$ is the momentum-sliced Hamiltonian with momentum *k_y_* and $\boldsymbol {r}=(x,z)$. We define Green’s function ${\mathbf {G}}^{r}_{k_{y}}(E)=\sum _{n}|\psi _{k_{y},n}\rangle \langle \psi _{k_{y},n}|/(E-E_{k_{y},n}+i0^{+})$, where |ψ_*n*_〉 is the *n*th eigenstate of $H(k_{y},\boldsymbol {r})$. We write ${\mathbf {G}}^{r}_{k_{y}}(E)$ in real-space form as $G^{r}_{k_{y}}(E,{\boldsymbol r},{\boldsymbol r}^{\prime })=\langle {\boldsymbol r} |{\mathbf {G}}^{r}_{k_{y}}(E)|{\boldsymbol r}^{\prime }\rangle$.

The velocity operator in the *y* direction for a given *k_y_* is $v_{y}(k_{y},\boldsymbol {r})=\partial H(k_{y},\boldsymbol {r})/\hbar \partial k_{y}$. The local current density in the *x*-*z* plane can be expressed as


(15) \begin{eqnarray*} J_{y}(E,\boldsymbol {r})=\int _{-\pi }^{\pi }\frac{dk_{y}}{2\pi }j_{y,k_{y}}(E,\boldsymbol {r}) \end{eqnarray*}

with the local current density for a given *k_y_* given by


(16) \begin{eqnarray*} &&j_{y,k_{y}}(E,\boldsymbol {r})\\ &&\quad =-\frac{1}{\pi }\frac{e}{\hbar }{\rm ImTr}\bigg [\frac{\partial H(k_{y})}{\partial k_{y}}G^{r}_{k_{y}}(E,\boldsymbol {r},\boldsymbol {r})\bigg ]. \\ \end{eqnarray*}

Equation (16) can be derived as follows:


(17) \begin{eqnarray*} &&j_{y,k_{y}}(E,\boldsymbol {r})\\ &&\quad =ev(k_{y},\boldsymbol {r})\rho (E,\boldsymbol {r}) \\ &&\quad =\sum _{n}ev_{n}(k_{y},\boldsymbol {r}) \delta (E-E_{k_{y},n}) \\ &&\quad =-\frac{1}{\pi }\frac{e}{\hbar }{\rm ImTr}\bigg [\frac{\partial H(k_{y})}{\partial k_{y}}G^{r}_{k_{y}}(E,\boldsymbol {r},\boldsymbol {r})\bigg ]. \\ \end{eqnarray*}

Here $\rho (E,{\boldsymbol r})$ is the local density of states at energy *E* and position ${\boldsymbol r}$, which can be expanded as $\rho (E,{\boldsymbol r})=\sum _{n}\delta (E-E_{k_{y},n})$.

## Supplementary Material

nwad025_Supplemental_File
