# Supplementary material for: Half-quantized helical hinge currents in axion insulators
Source: Natl Sci Rev. 2023 Feb 6;10(9):nwad025. doi: 10.1093/nsr/nwad025 (PMC10411682; doi:10.1093/nsr/nwad025)
Supplement: nwad025_Supplemental_File [file nwad025_supplemental_file.pdf]

Supplementary Materials for "Half-Quantized Helical Hinge Currents in Axion Insulators"

Ming Gong,[1] Haiwen Liu,[2] Hua Jiang,[3,4,*] Chui-Zhen Chen,[3,4,†] and X. C. Xie[1,5,‡]

[1]*International Center for Quantum Materials, School of Physics, Peking University, Beijing 100871, China*
[2]*Center for Advanced Quantum Studies, Department of Physics, Beijing Normal University, Beijing 100875, China*
[3]*School of Physical Science and Technology, Soochow University, Suzhou 215006, China.*
[4]*Institute for Advanced Study, Soochow University, Suzhou 215006, China.*
[5]*CAS Center for Excellence in Topological Quantum Computation, University of Chinese Academy of Sciences, Beijing 100190, China*

Sec1. STATIONARY PHASE METHOD IN CALCULATING THE GH SHIFT

We use the stationary phase method to derive the expression of the GH shift Δ_{GH} [1–3]. We consider the scattering problem described by the 2D Dirac Hamiltonian

$$\mathcal{H}(\boldsymbol{r}) = \begin{cases} \hbar v_F(-i\sigma_x\partial_x - i\sigma_y\partial_y) - U & (x \leqslant 0) \\ \hbar v_F(-i\sigma_x\partial_x - i\sigma_y\partial_y) + m\sigma_z & (x > 0) \end{cases} \tag{S1}$$

used in the main text. The incident plane wave and the reflected plane wave are $\psi_{\rm in}(\boldsymbol{r}) = e^{ik_xx+ik_yy}[e^{-i\frac{\alpha}{2}}, e^{i\frac{\alpha}{2}}]^T/\sqrt{2}$ and $\psi_{\rm re}(\boldsymbol{r}) = e^{-ik_xx+ik_yy}[e^{-i\frac{\pi-\alpha}{2}}, e^{i\frac{\pi-\alpha}{2}}]^T/\sqrt{2}$. The scattering problem is solved by matching the boundary conditions of incident/reflected wave $\psi(\boldsymbol{r}) = \psi_{\rm in} + r\psi_{\rm re}$ $(x \leqslant 0)$ and the penetrated evanescent wave $\psi_{\rm eva}(\boldsymbol{r}) = e^{-\kappa x+ik_yy}\psi(0,0)$ $(x > 0)$, and obtain the reflection coefficient $r = e^{i\phi_r}$. We have

$$r = e^{i\phi_r} = \frac{me^{-i\frac{\alpha}{2}} + i(\kappa - k_y)e^{i\frac{\alpha}{2}} - Ee^{-i\frac{\alpha}{2}}}{ime^{i\frac{\alpha}{2}} + (\kappa - k_y)e^{-i\frac{\alpha}{2}} - iEe^{i\frac{\alpha}{2}}}, \tag{S2}$$

where $\kappa = \sqrt{k_y^2 + m^2 - E^2}$, $E = \sqrt{k_x^2 + k_y^2} - U$, and $\alpha = \arctan\frac{k_y}{k_x}$. Therefore, ϕ_r can be viewed as a function of k_y or α for fixed E, U, and m.

The incident and reflected Gaussian wave packets are constructed as

$$\psi_g^{\rm in}(\boldsymbol{r}) = \int dk_y \frac{1}{\sqrt{2\pi}\Delta_{k_y}} \exp[-\frac{(k_y - \bar{k}_y)^2}{2\Delta_{k_y}^2}] e^{ik_xx+ik_yy} \frac{1}{\sqrt{2}}[e^{-i\frac{\alpha}{2}}, e^{i\frac{\alpha}{2}}]^T, \tag{S3}$$

$$\psi_g^{\rm re}(\boldsymbol{r}) = \int dk_y \frac{1}{\sqrt{2\pi}\Delta_{k_y}} \exp[-\frac{(k_y - \bar{k}_y)^2}{2\Delta_{k_y}^2}] e^{-ik_xx+ik_yy+i\phi_r} \frac{1}{\sqrt{2}}[e^{-i\frac{\pi-\alpha}{2}}, e^{i\frac{\pi-\alpha}{2}}]^T. \tag{S4}$$

Note that the integration is only performed for k_y because the eigen equation $\mathcal{H}\psi_g^{\rm in/re} = E\psi_g^{\rm in/re}$ constraints the number of free variables through $k_x = \sqrt{(E+U)^2 - k_y^2}$ and $\alpha = \arctan\frac{k_y}{k_x}$ are functions of k_y. Expand $\alpha(k_y)$ and $\phi_r(k_y)$ to the first order of $(k_y - \bar{k}_y)$ around $\bar{k}_y$ as $\alpha = \bar{\alpha} + (k_y - \bar{k}_y)\partial\alpha/\partial k_y + O((k_y - \bar{k}_y)^2)$ and $\phi_r = \bar{\phi}_r + (k_y - \bar{k}_y)\partial\bar{\phi}_r/\partial k_y + O((k_y - \bar{k}_y)^2)$. Then substitute them into Eq. (S3) and Eq. (S4) we have (accurate to the first order of k_y)

$$\psi_{g,\pm}^{\rm in}(\boldsymbol{r}) \propto e^{-(y \mp \frac{1}{2}\frac{\partial\bar{\alpha}}{\partial k_y})^2\Delta_{k_y}^2/2} \tag{S5}$$

$$\psi_{g,\pm}^{\rm re}(\boldsymbol{r}) \propto e^{-(y + \frac{\partial\bar{\phi}_r}{\partial k_y} \pm \frac{1}{2}\frac{\partial\bar{\alpha}}{\partial k_y})^2\Delta_{k_y}^2/2} \tag{S6}$$

for spin up (+) and spin down (−) components. From now on we replace $\bar{\phi}_r$, $\bar{k}_y$, and $\bar{\alpha}$ with ϕ_r, k_y, and α. It is clear from Eq. (S5) and Eq. (S6) that the GH shift is the displacement of the wave packet center. GH shifts for spin up and spin down components are $\Delta_{GH}^+ = -\partial\phi_r/\partial k_y - \partial\alpha/\partial k_y$ and $\Delta_{GH}^- = -\partial\phi_r/\partial k_y + \partial\alpha/\partial k_y$. The total GH shift is the spin averaged wave packet center displacement $\Delta_{GH} = (\Delta_{GH}^+ + \Delta_{GH}^-)/2 = -\frac{\partial\phi_r}{\partial k_y}$. Substitute it into Eq. (S2) we have

$$\Delta_{GH} = -\frac{\partial\phi_r}{\partial k_y} = 2\frac{m\kappa(m-E) + \kappa^2(k_y - \kappa) + (E-m)(k_y - \kappa)k_x\cos\alpha}{\kappa k_x[(E-m)^2 + (\kappa - k_y)^2 + 2(\kappa - k_y)(E-m)\sin\alpha]}. \tag{S7}$$

* jianghuaphy@suda.edu.cn
† czchen@suda.edu.cn
‡ xcxie@pku.edu.cn

Sec2. ANOMALOUS VELOCITY AND BAND MODIFICATION BY THE GH SHIFT

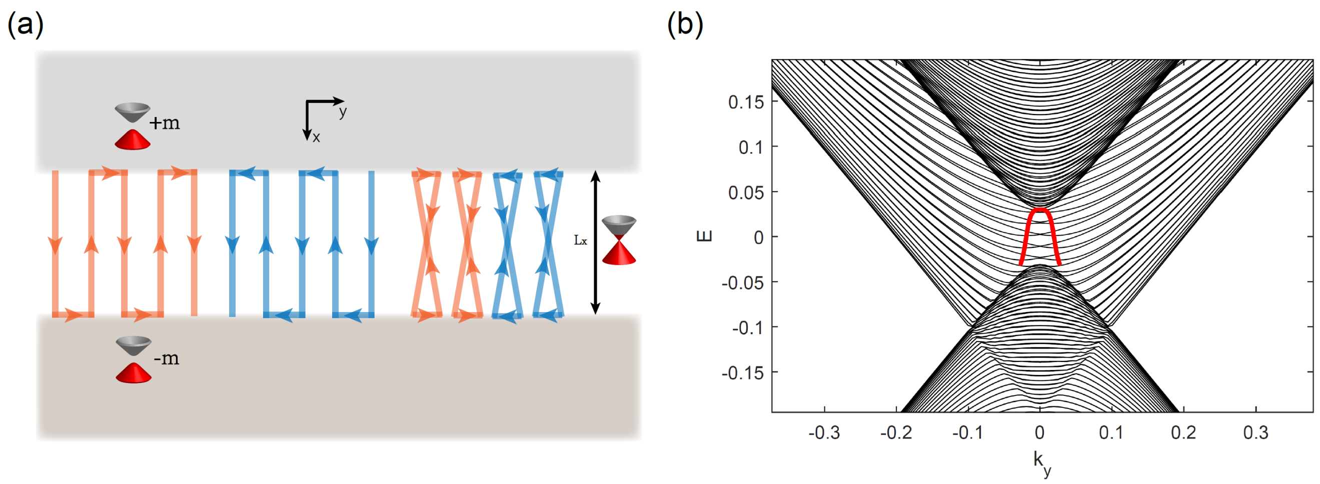

FIG. S1. (a) Sketch of the process where massless Dirac electron bounces back and forth between massive barriers with opposite mass. Trajectories colored blue and red represent electrons from difference valleys at $k_y = 0$ [$(0,0)$ and $(0,\pi)$]. (b) Band structure modification by the GH shift induced anomalous velocity. The band is numerically obtained from a tight binding model ($\mu = 0.2$, $L_x = 600$, and $m = 0.03$). The red curve represents the predicted band minimum calculated from the GH shift, which suggests the existence of the zero-velocity state at non-zero k_y.

Before deriving the half-quantized GH shift current in AIs and CIs, we investigate the anomalous velocity induced by the GH shift and its modification to the band structure. Consider a massless Dirac electron bounces back and forth between massive barriers with opposite mass as shown in Fig. S1(a). The average time interval between two consecutive bounces is $\Delta\tau = \frac{L_x}{v_F \cos\alpha}$. The anomalous velocity induced by the GH shift along the y direction is

$$v_{GH} = \frac{\Delta_{GH}}{\Delta\tau} = \frac{\Delta_{GH} v_F \cos\alpha}{L_x}, \quad (-\pi/2 < \alpha < \pi/2) \tag{S8}$$

For electrons with incident angle α, the drift velocity (without the GH shift induced anomalous velocity) is $v_0 = v_F \sin\alpha$. The total velocity vanishes at the band minimum $v_0 + v_{GH} = 0$. We then have the condition for the band minimum

$$-\frac{\Delta^y}{L_x \tan\alpha} = 1. \tag{S9}$$

The band minimum indicates the existence of the "8" shape trajectories as sketched in Fig. S1(a).

We use the 2D tight binding Hamiltonian [4]

$$H_{2D} = \sum_{\mathbf{i}} \Big[\frac{i\hbar v_F}{2a} (c^\dagger_{\mathbf{i}} \sigma_y c_{\mathbf{i}+\delta\hat{\mathbf{x}}} - c^\dagger_{\mathbf{i}} \sigma_x c_{\mathbf{i}+\delta\hat{\mathbf{y}}}) - \frac{\mu}{2} c^\dagger_{\mathbf{i}} \sigma_0 c_{\mathbf{i}} + m c^\dagger_{\mathbf{i}} \sigma_z c_{\mathbf{i}} \Big] + \text{H.c.} \tag{S10}$$

to numerically investigate the band structure changes. We take y direction to be infinite and in the x direction the massless electrons with $m = 0, \mu \neq 0$ sandwiched between two barriers with opposite m and $\mu = 0$. The lattice Hamiltonian described by Eq. (S10) contains 4 valleys in total at $(0,0)$, $(0,\pi)$, $(\pi,0)$, and (π,π). We investigate the two valleys at $k_y = 0$ [$(0,0)$ and $(0,\pi)$]. Electrons from both valleys accumulate anomalous velocity when they bounce off the massive barrier, but with opposite direction as sketched in Fig. S1(a). From Fig. S1(b) we can see that band minimums for electrons from different valleys splits, which coincide with the analytical prediction based on Eq. (S9).

Sec3. DERIVATION OF THE HALF-QUANTIZED GH SHIFT CURRENT

We derive the half-quantized hinge current induced by the GH shift. Suppose the width between the barriers is L_x, the average time between two consecutive bounces off one of the barriers is $\Delta\tau = 2L_x/v_F|\cos\alpha|$ for $-\pi < \alpha \leqslant \pi$. (Here, we consider the time interval between two consecutive bounces of electrons off the same barrier, thus the distance traveled in the x direction is

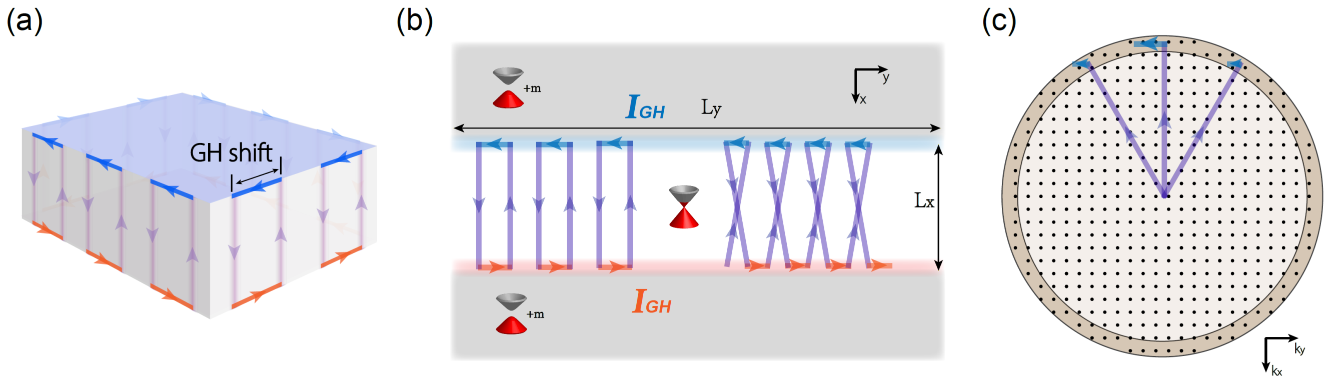

FIG. S2. (a) Sketch of the GH shift current induced chiral hinge current in the AI. (b) Expanded view of the top-side-bottom surfaces of the AI. Massless Dirac electrons bounce back and forth between massive barriers with the same mass m, accumulate an anomalous shift current due to the chiral GH shift. L_x and L_y represent the width and length of the side surface for box normalization (in calculations we take the y direction to be periodic). (c) Box normalization to count the GH shift contributions from electrons near the Fermi surface.

$2L_x$. Contributions from electrons with incident angle α and $\pi-\alpha$ are considered to be equivalent between successive bounces, i. e. $\Delta_{GH}(\alpha)=\Delta_{GH}(\pi-\alpha)$, since the electron with incident angle α will be alternated to $\pi-\alpha$ after one reflection. Therefore, we take $|\cos\alpha|$ to describe both cases.) The lateral GH shift Δ_{GH} induces an anomalous velocity of electrons near the barrier as $v_{GH}=\Delta_{GH}/\Delta\tau=\Delta_{GH}v_F|\cos\alpha|/2L_x(-\pi<\alpha\leqslant\pi)$. The total GH shift current is obtained by counting the contributions of all filled states as

$$I_{GH}=\sum_{\text{filled}}\frac{ev_{GH}}{L_y}=\sum_{\text{filled}}\frac{e\Delta_{GH}v_F|\cos\alpha|}{2L_xL_y}. \tag{S11}$$

Here, we take the box normalization for electrons on the side surface with length L_y and width L_x, as depicted in Fig. S2(b). According to the box normalization [Fig. S2(c)], $\frac{1}{L_xL_y}\sum_{\boldsymbol{k}}=\frac{1}{(2\pi)^2}\int d\boldsymbol{k}$.

$$I_{GH}=\int_{\text{filled}}\frac{L_xL_y}{(2\pi)^2}KdKd\alpha\frac{e\Delta_{GH}(\alpha)v_F|\cos\alpha|}{2L_xL_y} \tag{S12}$$

$$=\int_{-\infty}^{E_F}\frac{dE}{\hbar v_F}\int_{-\pi}^{\pi}\frac{Kd\alpha|\cos\alpha|}{(2\pi)^2}\frac{e\Delta_{GH}(\alpha)v_F}{2} \tag{S13}$$

$$=\int_{-\infty}^{E_F}dE\cdot 2\int_{-K}^{K}\frac{dk_y}{2\pi}\frac{e\Delta_{GH}(k_y,E)}{2\cdot 2\pi\hbar}$$

$$=\int_{-\infty}^{E_F}dE\frac{e}{h}\int_{-K}^{K}\frac{dk_y}{2\pi}\Delta_{GH}(k_y,E). \tag{S14}$$

From Eq. (S12) to Eq. (S13), we used the fact that $\int_{-\pi}^{\pi}\Delta_{GH}(\alpha)|\cos\alpha|d\alpha=2\int_{-\pi/2}^{\pi/2}\Delta_{GH}(\alpha)\cos\alpha d\alpha=2\int_{-\pi/2}^{\pi/2}\Delta_{GH}(\alpha)d\sin\alpha$ since we take $\Delta_{GH}(\alpha)=\Delta_{GH}(\pi-\alpha)$. $K=(E+U)/\hbar v_F$ is the Fermi wave vector at energy E.

Some points should be noted for Eq. (S13) and Eq. (S14). In Eq. (S13), the integrand $v_F\Delta_{GH}\cos\alpha$ is exactly the GH shift induced probability flux J_{GH}. From the flux conservation perspective $J_{GH}=J_{eva}+J_{int}-J_d$, the GH shift current have both contributions from the evanescent wave and the interference wave, indicating that our derivations of the GH shift as well as the GH shift current are equivalent. In Eq. (S14), the integration over E is not necessarily performed from $-\infty$ to E_F, since we only derived the Δ_{GH} for the total reflection case within the gap. The only thing we are interested is the differential GH shift current with respect to E_F, which contributes to the transport current. From Eq. (S7) $\Delta_{GH}=-\partial\phi_r/\partial k_y$ and we have the differential form of I_{GH}

$$\delta I_{GH}=\delta E_F\frac{e}{h}\int_{-K}^{K}\frac{dk_y}{2\pi}\Delta_{GH}(k_y,E)=\delta E_F\frac{e}{h}[\phi_r(-\pi/2)-\phi_r(\pi/2)]/2\pi \tag{S15}$$

for E_F lying in the gap of the top and bottom surfaces. From the plot in Fig. 2(c) in the main text, we immediately obtain the half-quantized GH shift current $\delta I_{GH}=\frac{e}{2h}\delta E_F$.

Sec4. POWER LAW DECAY OF THE INTERFERENCE WAVE PART OF THE GH SHIFT CURRENT

In the maintext we discussed the decomposition of the GH shift current according to the contributions from the evanescent wave part and the interference wave part. In this section, we emphasize that the interference wave induced GH shift current component decays from the edge following the power law, which is in stark contrast to the current carried by the topological edge or hinge state that decays exponentially. The y-component of the current as a function of x carried by the interference wave is $j_{int}(x) = \psi^{\dagger}_{\text{int}} v_F \sigma_y \psi_{\text{int}}(x) = 2v_F[\cos(\phi_r - 2k_x x) + \sin\alpha]$, where $k_x = K\cos\alpha$ and $K = (E_F + U)/\hbar v_F$ is the Fermi wave vector. The total contribution of the current from all the α can be written as

$$\begin{aligned}\mathcal{J}_{int}(x) &= \int_{-\frac{\pi}{2}}^{\frac{\pi}{2}} j_{int}(x)d\alpha \propto \int_{-\frac{\pi}{2}}^{\frac{\pi}{2}} [\cos(\phi_r - 2k_x x) + \sin\alpha]d\alpha \\ &= \int_{-\frac{\pi}{2}}^{\frac{\pi}{2}} \cos(\phi_r - 2k_x x)d\alpha. \end{aligned} \tag{S16}$$

From Eq. (S2), if $m \to \infty$, then $\phi_r \to 0$ for most of the α. We omit the ϕ_r for simplicity to study the asymptotic behavior of Eq. (S16). Then we have

$$\mathcal{J}_{int}(x) \propto \int_{-\frac{\pi}{2}}^{\frac{\pi}{2}} \cos(2k_x x)d\alpha = \frac{1}{2}\int_{0}^{2\pi} \cos(2K\cos\alpha x)d\alpha = \frac{1}{4\pi}\mathrm{J}_0(2Kx). \tag{S17}$$

Here, $\mathrm{J}_0(x)$ is the 0_{th} Bessel function. According to the asymptotic formula of the Bessel function

$$\begin{aligned}\mathrm{J}_n(x) &= \frac{1}{\pi}\mathrm{Re}\left[e^{-i\frac{n\pi}{2}}\int_{-\frac{\pi}{2}}^{\frac{\pi}{2}} e^{ix\cos\varphi}\cos n\varphi \mathrm{d}\varphi\right] \\ &= \sqrt{\frac{2}{\pi x}}\cos\left(x - \frac{n\pi}{2} - \frac{\pi}{4}\right) + O(x^{-\frac{3}{2}}),\end{aligned} \tag{S18}$$

one can see that when $x \to \infty$

$$\mathcal{J}_{int}(x) \propto \mathrm{J}_0(2Kx) \to \sqrt{\frac{1}{\pi K x}}\cos\left(2Kx - \frac{\pi}{4}\right). \tag{S19}$$

The result clearly shows that the interference wave part of the GH shift current maximizes at the boundary and decays to zero in a power law $x^{-\frac{1}{2}}$ when moving away from the boundary with the oscillation length as $1/2K$.

Here we emphasize that the chiral current carried by the interference wave on the metallic side surface depends on the gapped, time-reversal symmetry breaking top/bottom surface. The power low decay of the current induced by the interference wave indicates that it cannot be generated by any kind of topologically protected edge or hinge state which decays exponentially. It also indicates that the half-quantized hinge current cannot exist by itself and should be combined with another one to form quantized side surface transport in the AI or the CI. These features are unique for the half-quantized hinge current.

Sec5. DERIVATION OF THE DIFFERENTIAL CONDUCTANCE AND THE LOCAL CURRENT DENSITY

In this section, we derive the differential conductance and the local current density with the help of the non-equilibrium Green's function method [5–7]. As depicted in Fig. S3(c), we first consider the simplest case where the central region connects to an external lead. H_C and H_{Lead} are the Hamiltonians of the central region and the lead, H_I is the coupling between the central region and the lead. We use $a_{\mathbf{i}'}$ to denote the annihilation operator in the central region at site $\mathbf{i}'$ and $c_{\mathbf{i}}$ to denote the annihilation operator in the lead at site $\mathbf{i}$. The coupling Hamiltonian reads

$$H_I = t_{\mathbf{ii}'}c^{\dagger}_{\mathbf{i}}a_{\mathbf{i}'} + t^{*}_{\mathbf{i}'\mathbf{i}}a^{\dagger}_{\mathbf{i}'}c_{\mathbf{i}}. \tag{S20}$$

The particle leakage on the lead is

$$-\sum_{\mathbf{i}}\frac{dN_{\mathbf{i}}}{dt} = -\sum_{\mathbf{i}}\frac{d}{dt}\langle c^{\dagger}_{\mathbf{i}}c_{\mathbf{i}}\rangle = -\sum_{\mathbf{i}}\frac{1}{i\hbar}\langle[c^{\dagger}_{\mathbf{i}}c_{\mathbf{i}}, H]\rangle \tag{S21}$$

$$= -\sum_{\mathbf{ii}'}\frac{1}{i\hbar}\langle t_{\mathbf{ii}'}c^{\dagger}_{\mathbf{i}}a_{\mathbf{i}'} + t^{*}_{\mathbf{i}'\mathbf{i}}a^{\dagger}_{\mathbf{i}'}c_{\mathbf{i}}\rangle, \tag{S22}$$

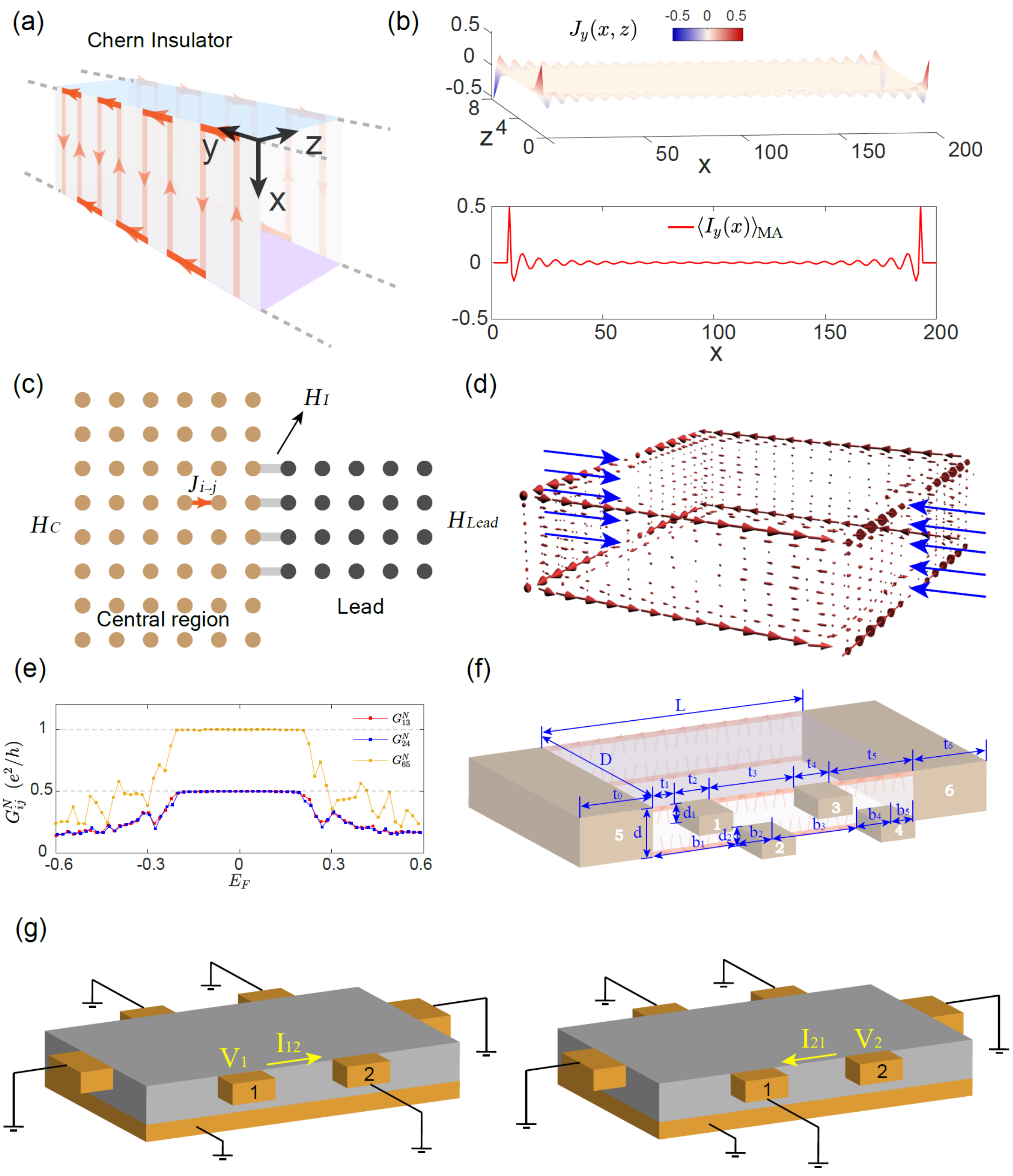

FIG. S3. (a) Schematic of the side surface hinge current in the CI. (b) The upper panel shows the distribution of $J_y(x,z)$ in the $x-z$ plane. The lower panel shows the moving averaged flux $\langle I_y(\bar{x})\rangle_{\rm MA}$ through the window $[\bar{x}-7 \leqslant x \leqslant \bar{x}+7, 0 \leqslant z \leqslant 4]$. Both current density J_y and flux I_y are in units of $e/h\Delta E$. (c) Schematic diagram showing how to calculate the local current density distributions. The central region described by the Hamiltonian H_C is connected to a external lead H_{Lead} through the coupling Hamiltonian H_I. $J_{i\to j}$ denotes the local current from site i to site j. (d) Local current distribution of the CI. (e) The nonreciprocal conductance $G^N_{ij} = G_{ij} - G_{ji}$ in the CI. (f) Schematic of the six-terminal device. Leads 5 and 6 connect to the ends of the CI film. Terminals 1~4 are surface leads with leads 1 and 3 (2 and 4) connected to the top (bottom) surface of the sample. (f) Schematic of the six-terminal device and the illustration of the relationship between the quantized chiral edge transport and the half-quantized hinge transport in the CI phase. (g) Schematic of the experimental setup to measure the nonreciprocal conductances G^N_{ij} between leads. When measuring G_{12}, the voltage V_1 is applied to lead 1 with all the other leads grounded and collect the current I_{12} flowing into lead 2. The conductance is calculated through $G_{12} = I_{12}/V_1$. Then measure $G_{21} = I_{21}/V_2$. The difference between G_{12} and G_{21} gives G^N_{12}.

where $H = H_C + H_{Lead} + H_I$. From Eq. (S21) to Eq. (S22) we used the relation $[H_C, \sum_{\mathbf{i}} N_{\mathbf{i}}] = [H_{Lead}, \sum_{\mathbf{i}} N_{\mathbf{i}}] = 0$. Through relations

$$\begin{aligned} i\langle c^\dagger_{\mathbf{i}}(t=0)a_{\mathbf{i}'}(t=0)\rangle &= G^<_{\mathbf{i}'\mathbf{i}}(t=0) = \int_{-\infty}^{\infty} \frac{dE}{2\pi} G^<_{\mathbf{i}'\mathbf{i}}(E) \\ i\langle a^\dagger_{\mathbf{i}'}(t=0)c_{\mathbf{i}}(t=0)\rangle &= G^<_{\mathbf{i}\mathbf{i}'}(t=0) = \int_{-\infty}^{\infty} \frac{dE}{2\pi} G^<_{\mathbf{i}\mathbf{i}'}(E). \end{aligned} \tag{S23}$$

Combine Eq. (S22) and Eq. (S23), we obtain the net current flowing into the lead as

$$I_{Lead} = -e\sum_{\mathbf{i}} \frac{dN}{dt} = \frac{e}{\hbar}\sum_{\mathbf{i}\mathbf{i}'} \int_{-\infty}^{\infty} \frac{dE}{2\pi}[t_{\mathbf{i}\mathbf{i}'} G^<_{\mathbf{i}'\mathbf{i}}(E) - t^*_{\mathbf{i}'\mathbf{i}} G^<_{\mathbf{i}\mathbf{i}'}(E)]. \tag{S24}$$

We denote the Green's function of the free lead (without coupling to the central region) as $\mathbf{g}$, then use the Langreth theorem and Dyson equations we have

$$G^<_{\mathbf{i}'\mathbf{i}} = \sum_{\mathbf{j}\mathbf{j}'} (G_{\mathbf{i}'\mathbf{j}'} t^*_{\mathbf{j}'\mathbf{j}} g_{\mathbf{j}\mathbf{i}})^< = \sum_{\mathbf{j}\mathbf{j}'} [G^r_{\mathbf{i}'\mathbf{j}'} t^*_{\mathbf{j}'\mathbf{j}} g^<_{\mathbf{j}\mathbf{i}} + G^<_{\mathbf{i}'\mathbf{j}'} t^*_{\mathbf{j}'\mathbf{j}} g^a_{\mathbf{j}\mathbf{i}}] \tag{S25}$$

$$G^<_{\mathbf{i}\mathbf{i}'} = \sum_{\mathbf{j}\mathbf{j}'} (g_{\mathbf{i}\mathbf{j}} t^*_{\mathbf{j}\mathbf{j}'} G_{\mathbf{j}'\mathbf{i}'})^< = \sum_{\mathbf{j}\mathbf{j}'} [g^r_{\mathbf{i}\mathbf{j}} t^*_{\mathbf{j}\mathbf{j}'} G^<_{\mathbf{j}'\mathbf{i}'} + g^<_{\mathbf{i}\mathbf{j}} t^*_{\mathbf{j}\mathbf{j}'} G^a_{\mathbf{j}'\mathbf{i}'}]. \tag{S26}$$

Then we have

$$I_{Lead} = \frac{e}{\hbar}\sum_{\mathbf{i}\mathbf{i}'\mathbf{j}\mathbf{j}'} \int_{-\infty}^{\infty} \frac{dE}{2\pi}(t_{\mathbf{i}\mathbf{i}'} G^r_{\mathbf{i}'\mathbf{j}'} t^*_{\mathbf{j}'\mathbf{j}} g^<_{\mathbf{j}\mathbf{i}} + t_{\mathbf{i}\mathbf{i}'} G^<_{\mathbf{i}'\mathbf{j}'} t^*_{\mathbf{j}'\mathbf{j}} g^a_{\mathbf{j}\mathbf{i}} - t_{\mathbf{i}'\mathbf{i}} g^r_{\mathbf{i}\mathbf{j}} t^*_{\mathbf{j}\mathbf{j}'} G^<_{\mathbf{j}'\mathbf{i}'} - t_{\mathbf{i}'\mathbf{i}} g^<_{\mathbf{i}\mathbf{j}} t^*_{\mathbf{j}\mathbf{j}'} G^a_{\mathbf{j}'\mathbf{i}'}). \tag{S27}$$

We define self-energies

$$\Sigma^{</r/a}_{\mathbf{j}'\mathbf{i}'} = \sum_{\mathbf{i}\mathbf{j}} t^*_{\mathbf{j}'\mathbf{j}} g^{</r/a}_{\mathbf{j}\mathbf{i}} t_{\mathbf{i}\mathbf{i}'}, \tag{S28}$$

and substitute it into Eq. (S27) as

$$\begin{aligned} I_{Lead} &= \frac{e}{\hbar}\int_{-\infty}^{\infty} \frac{dE}{2\pi}(G^r_{\mathbf{i}'\mathbf{j}'}\Sigma^<_{\mathbf{j}'\mathbf{i}'} + G^<_{\mathbf{i}'\mathbf{j}'}\Sigma^a_{\mathbf{j}'\mathbf{i}'} - G^<_{\mathbf{j}'\mathbf{i}'}\Sigma^r_{\mathbf{i}'\mathbf{j}'} - G^a_{\mathbf{j}'\mathbf{i}'}\Sigma^<_{\mathbf{i}'\mathbf{j}'}) \\ &= \frac{e}{\hbar}\int_{-\infty}^{\infty} \frac{dE}{2\pi}[\Sigma^<_{\mathbf{i}'\mathbf{j}'}(G^r_{\mathbf{j}'\mathbf{i}'} - G^a_{\mathbf{j}'\mathbf{i}'}) + G^<_{\mathbf{i}'\mathbf{j}'}(\Sigma^a_{\mathbf{j}'\mathbf{i}'} - \Sigma^r_{\mathbf{j}'\mathbf{i}'})]. \end{aligned} \tag{S29}$$

According to the fluctuation-dissipation theorem

$$g^<_{\mathbf{j}\mathbf{i}} = i f_{Lead} A^<_{\mathbf{j}\mathbf{i}} = -f_{Lead}(g^r_{\mathbf{j}\mathbf{i}} - g^a_{\mathbf{j}\mathbf{i}}), \tag{S30}$$

where $\mathbf{A}^< = i(\mathbf{g}^r - \mathbf{g}^a)$ is the spectral function and $f_{Lead} = 1/(e^{(E-\mu_n)/k_B T} + 1)$ is the Fermi distribution function of the lead. We then have

$$\Sigma^<_{\mathbf{i}'\mathbf{j}'} = -f_{Lead}[\Sigma^r_{\mathbf{i}'\mathbf{j}'} - \Sigma^a_{\mathbf{i}'\mathbf{j}'}]. \tag{S31}$$

Define the linewidth function $\Gamma_{Lead,\mathbf{i}'\mathbf{j}'} = i(\Sigma^r_{\mathbf{i}'\mathbf{j}'} - \Sigma^a_{\mathbf{i}'\mathbf{j}'})$ and substitute it into Eq. (S29)

$$\begin{aligned} I_{Lead} &= \frac{e}{\hbar}\int_{-\infty}^{\infty} \frac{dE}{2\pi}[i\Gamma_{Lead,\mathbf{i}'\mathbf{j}'} f_{Lead}(G^r_{\mathbf{j}'\mathbf{i}'} - G^a_{\mathbf{j}'\mathbf{i}'}) + i\Gamma_{Lead,\mathbf{i}'\mathbf{j}'} G^<_{\mathbf{i}'\mathbf{j}'}] \\ &= \frac{ie}{\hbar}\int_{-\infty}^{\infty} \frac{dE}{2\pi}\mathrm{Tr}[f_{Lead}\mathbf{\Gamma}_{Lead}(\mathbf{G}^r - \mathbf{G}^a) + \mathbf{\Gamma}_{Lead}\mathbf{G}^<]. \end{aligned} \tag{S32}$$

Now we consider a multi-terminal system. We use $\mathbf{\Gamma}_n$ and f_n to denote the linewidth function and the Fermi distribution function of the n_{th} lead. Define the total self-energy from all the leads

$$\mathbf{\Sigma}^< = \sum_n -f_n(\mathbf{\Sigma}^r - \mathbf{\Sigma}^a) = \sum_n i f_n \mathbf{\Gamma}_n. \tag{S33}$$

According to the Keldysh formula we have

$$\mathbf{G}^< = \mathbf{G}^r\mathbf{\Sigma}^<\mathbf{G}^a = \sum_n \mathbf{G}^r i f_n \mathbf{\Gamma}_n \mathbf{G}^a. \tag{S34}$$

The Dyson equations $\mathbf{G}^{r/a} = \mathbf{g}^{r/a} + \mathbf{g}^{r/a}\mathbf{\Sigma}^{r/a}\mathbf{G}^{r/a}$ implies $[\mathbf{g}^{r/a}]^{-1} = [\mathbf{G}^{r/a}]^{-1} + \mathbf{\Sigma}^{r/a}$. Note that $[\mathbf{g}^{r}]^{-1} = [\mathbf{g}^{a}]^{-1} = E - \mathbf{H}_{Lead}$, therefore $[\mathbf{G}^{a}]^{-1} - [\mathbf{G}^{r}]^{-1} = \mathbf{\Sigma}^{r} - \mathbf{\Sigma}^{a} = \sum_n -i\mathbf{\Gamma}_n$. Then we have

$$\mathbf{G}^r - \mathbf{G}^a = \sum_n -i\mathbf{G}^r\mathbf{\Gamma}_n\mathbf{G}^a. \tag{S35}$$

Combine Eq. (S32), Eq. (S34), and Eq. (S35), we obtain the current flowing into the $m_{\rm th}$ terminal as

$$\begin{aligned} I_m =& \frac{e}{\hbar}\int_{-\infty}^{\infty}\frac{dE}{2\pi}\sum_n \mathrm{Tr}[\mathbf{\Gamma}_m f_m \mathbf{G}^r\mathbf{\Gamma}_n\mathbf{G}^a - \mathbf{\Gamma}_m\mathbf{G}^r f_n\mathbf{\Gamma}_n\mathbf{G}^a] \\ =& \frac{e}{\hbar}\int_{-\infty}^{\infty}\frac{dE}{2\pi}\sum_n (f_m - f_n)\mathrm{Tr}[\mathbf{\Gamma}_m\mathbf{G}^r\mathbf{\Gamma}_n\mathbf{G}^a]. \end{aligned} \tag{S36}$$

At zero temperature, the Fermi distribution function becomes $f_n(E) = \Theta(E_{F,n} - E) = \Theta(-eV_n - E)$, where $\Theta(x)$ is the Heaviside step function and V_n is the gate voltage applied to the terminal n. For small bias we take the approximation $\int_{-\infty}^{\infty} dE(f_m - f_n)(\cdots) \approx (E_{F,m} - E_{F,n})(\cdots) = e(V_n - V_m)(\cdots)$ and we have

$$I_m = \frac{e^2}{h}\sum_n (V_n - V_m)T_{mn} = G_{mn}(V_m - V_n), \tag{S37}$$

where the differential conductance $G_{mn} = \frac{e^2}{h}T_{mn}$ and the transmission coefficient $T_{mn} = \mathrm{Tr}[\mathbf{\Gamma}_m\mathbf{G}^r\mathbf{\Gamma}_n\mathbf{G}^a]$.

We now derive the local current density distribution [5–8]. Consider the local Hamiltonian on a given site $\mathbf{i}$ as

$$H_I = \sum_{\mathbf{j}}(H_{\mathbf{ji}}c_{\mathbf{j}}^{\dagger}c_{\mathbf{i}} + H_{\mathbf{ij}}c_{\mathbf{i}}^{\dagger}c_{\mathbf{j}}). \tag{S38}$$

The current flowing into the site $\mathbf{i}$ is

$$\begin{aligned} I_{\mathbf{i}} =& -e\langle \dot{N}_{\mathbf{i}}\rangle = -\frac{e}{i\hbar}\langle [N_{\mathbf{i}}, H_I]\rangle \\ =& -\frac{e}{i\hbar}\langle [c_{\mathbf{i}}^{\dagger}c_{\mathbf{i}}, \sum_{\mathbf{j}} H_{\mathbf{ji}}c_{\mathbf{j}}^{\dagger}c_{\mathbf{i}} + H_{\mathbf{ij}}c_{\mathbf{i}}^{\dagger}c_{\mathbf{j}}]\rangle \\ =& -\frac{e}{i\hbar}\langle \sum_{\mathbf{j}} -H_{\mathbf{ji}}c_{\mathbf{j}}^{\dagger}c_{\mathbf{i}} + H_{\mathbf{ij}}c_{\mathbf{i}}^{\dagger}c_{\mathbf{j}}\rangle \\ =& \sum_{\mathbf{j}} J_{\mathbf{j}\to\mathbf{i}}. \end{aligned} \tag{S39}$$

The local current from site $\mathbf{j}$ to $\mathbf{i}$ [see Fig. S3(c)] can further be expressed as

$$\begin{aligned} J_{\mathbf{j}\to\mathbf{i}} =& -\frac{e}{i\hbar}(-H_{\mathbf{ji}}\langle c_{\mathbf{j}}^{\dagger}c_{\mathbf{i}}\rangle + H_{\mathbf{ij}}\langle c_{\mathbf{i}}^{\dagger}c_{\mathbf{j}}\rangle) \\ =& -\frac{e}{i\hbar}(iH_{\mathbf{ji}}G_{\mathbf{ij}}^{<} - iH_{\mathbf{ij}}G_{\mathbf{ji}}^{<}) \\ =& -\frac{2e}{\hbar}\int_{-\infty}^{\infty}\frac{dE}{2\pi}\mathrm{ReTr}[H_{\mathbf{ji}}G_{\mathbf{ij}}^{<}(E)]. \end{aligned} \tag{S40}$$

With the help of Eq. (S33) and Eq. (S34), the above expression can be written as

$$\begin{aligned} J_{\mathbf{j}\to\mathbf{i}} =& -\frac{2e}{\hbar}\int_{-\infty}^{\infty}\frac{dE}{2\pi}\mathrm{ReTr}[H_{\mathbf{ji}}\sum_n G_{\mathbf{ii'}}^{r}(E)\Gamma_{n,\mathbf{i'j}}(E)f_n G_{\mathbf{j'j}}^{a}(E)] \\ =& -\frac{2e}{\hbar}\int_{-\infty}^{\infty}\frac{dE}{2\pi}\sum_n \mathrm{ImTr}[H_{\mathbf{ji}}(\mathbf{G}^r(E)f_n\mathbf{\Gamma}_n(E)\mathbf{G}^a(E))_{\mathbf{ij}}]. \end{aligned} \tag{S41}$$

At zero temperature, the Fermi distribution function becomes $f_n(E) = \Theta(E_{F,n} - E) = \Theta(-eV_n - E)$ and the integral becomes $\int_{-\infty}^{\infty} dEf_n(\cdots) = \int_{-\infty}^{-eV_n} dE(\cdots)$. Then it is straightforward to obtain

$$\begin{aligned} J_{\mathbf{j}\to\mathbf{i}} =& -\frac{2e}{h}\sum_n\int_{-\infty}^{-eV_n} dE\,\mathrm{ImTr}[H_{\mathbf{ji}}(\mathbf{G}^r(E)\mathbf{\Gamma}_n(E)\mathbf{G}^a(E))_{\mathbf{ij}}] \\ =& -\frac{2e}{h}\sum_n\int_{-\infty}^{0} dE\,\mathrm{ImTr}[H_{\mathbf{ji}}(\mathbf{G}^r(E)\mathbf{\Gamma}_n(E)\mathbf{G}^a(E))_{\mathbf{ij}}] - \frac{2e}{h}\sum_n\int_{0}^{-eV_n} dE\,\mathrm{ImTr}[H_{\mathbf{ji}}(\mathbf{G}^r(E)\mathbf{\Gamma}_n(E)\mathbf{G}^a(E))_{\mathbf{ij}}]. \end{aligned} \tag{S42}$$

The first term in Eq. (S42) represents the equilibrium current while the second term represents the non-equilibrium transport current. For small bias, the non-equilibrium transport current can be simplified as

$$J_{neq,\mathbf{j}\to\mathbf{i}} = -\frac{2e}{h}\sum_n V_n \mathrm{ImTr}[H_{\mathbf{ji}}(\mathbf{G}^r(E)\mathbf{\Gamma}_n(E)\mathbf{G}^a(E))_{\mathbf{ij}}]. \tag{S43}$$

Sec6. CHIRAL EDGE TRANSPORT AND ITS RELATION TO HALF-QUANTIZED HINGE CHANNELS IN CHERN INSULATORS

The cross-section local current density for the CI is shown in Fig. S3(a) and (b). When side surface electrons bounce back and forth between the top and bottom surfaces, the direction of the GH shift currents on the two hinges flow in the same direction, giving rise to chiral net side surface current. Fig. S3(b) shows the spatial distribution of the cross-section local current density for the CI. Similar to the AI case as analyzed in the main text, the local current peaks at the hinges, but the hinge currents flow in the same direction, leading to a net chiral side surface current which is in sharp distinction from the AI. In the upper panel of Fig. S3(d) we also plot the local transport current distribution calculated by Eq. (S43). Here, the CI bulk is connected to two external leads as depicted by the blue arrows in the upper panel of Fig. S3(d) and we take $V_1 = V_2$ to investigate the chiral or helical nature of the side surface current. In Fig. S3(e) we demonstrate that the nonreciprocal conductances on the top and bottom hinges of the CI have the same sign, contributing to totally quantized side surface transport. In Fig. S3(f) we illustrate that the quantized chiral conductance channel in the CI originates from the combination of the two half-quantized hinge channels $(1/2 + 1/2)$. The quantized chiral edge transport in the CI can also be understood from a adiabatic charge pump point of view. According to Eq. (2.6) in [9], the adiabatic charge pump is $c_n = -e\int_0^T dt \int_{BZ} \Omega^n_{qt} dq/2\pi$, where T denotes the period of the cyclic pump and q denotes the momentum. Similarly, in our model the net charge pump during the reflection process is

$$c = -e\int_{-E/\hbar v_F}^{E/\hbar v_F} \frac{dk_y}{2\pi}\int_{-T(k_y)}^{T(k_y)} dt\Omega_{k_y t} = \mathrm{sgn}(m)\frac{e}{2}, \tag{S44}$$

indicating that the net charge pump for the reflection on one of the massive barrier is exactly half-charge $\frac{e}{2}$. When we take the other massive barrier to describe the side surface of the AI or the CI, the total charge pumped along the y direction is 0 $(1/2-1/2)$ in the AI and e $(1/2 + 1/2)$ in the CI. The quantized e charge pump indicates the existence of the quantized chiral edge channel in CIs.

Sec7. EXPERIMENTAL SETUPS TO MEASURE THE NONRECIPROCAL CONDUCTANCES

In this section, we illustrate the principles in measuring the nonreciprocal conductances in multi-terminal devices. We consider the nonreciprocal conductances between two external leads [such as lead 1 and lead 2 as shown in Fig. S3(g)]. To obtain G^N_{12}, the conductances G_{12} and G_{21} should be measured. G_{12} is defined as $G_{12} = I_{12}/V_1$, where V_1 is the applied gate voltage on lead 1 with all the other leads grounded as shown in Fig. S3(g), and I_{12} is the current flowing into lead 2. Here, we only consider the differential conductances so that the I_{12} is the differential current induced by the small bias V_1. The measurement of $G_{21} = I_{21}/V_2$ is similar.

In experiments, fabricating the multi-terminal device in Fig. S3(g) (the bottom surface of the sample is grounded and all the other leads are connected near the top surface of the sample) may be easier than the six-terminal device shown in Fig. S3(f) [10]. Moreover, since the bottom surface of the sample is grounded in Fig. S3(f), the half-quantized hinge channel localized on the bottom hinge of the AI or CI is inactive, thus only the half-quantized hinge channel on the top hinge contributes to the nonreciprocal conductance. Therefore, the measurement result of G^N_{12} is sensitive to the quality of lead 1 and lead 2. To improve the experimental accuracy, we emphasize that the surface leads [lead 1 and lead 2 in Fig. S3(f)] should be thick enough (but do not touch the bottom surface) such that they couple to more conducting side surface channels. Besides, lead 1 and lead 2 should be close enough to ensure that nearly all hinge current can flow into the measuring lead.

Sec8. MODEL PARAMETERS IN NUMERICS

In Sec2, the parameters in the Hamiltonian Eq. (S10) are $\hbar v_F/a = 1$. For the gapless region $\mu = 0.2$ and $m = 0$, for the gapped region $\mu = 0$ and $m = 0.03$. $L_x = 600$.

In Sec6 and the main text, in calculating the cross-section local current density, we take the parameters of the 3D TI Hamiltonian as $A = 1$, $B = 0.6$, $M_0 = 1$, $E_F = 0.4$ and $M = 0.6$. In the semi-magnetic TI case, the magnetization term $H_M = 0.6\times\tau_0\otimes\sigma_z$ only couples to the top surface, while in the AI/CI case $H_M = \pm 0.6\times\tau_0\otimes\sigma_z$ couple to the top and bottom surfaces.

In calculating the local current distributions we take $A = 1$, $B = 0.6$, $M_0 = 1$, $E_F = 0.4$ and $M = 0.6$. The system size (for both AI and CI) is $15 \times 15 \times 30$. In plotting Fig. S3(d) and the Fig. 5(b) in the main text, the thickness is squeezed but does not affect the results in demonstrating the helical/chiral transport nature on the AI/CI side surface.

In calculating the nonreciprocal conductances, we take $A = 1$, $B = 0.6$, $M_0 = 1$, $E_F = 0.4$ and $M = 0.2$. The geometrical size of the six-terminal device is marked in Fig. S3(e). We take $t_0 = 25$, $t_1 = 1$, $t_2 = 11$, $t_3 = 1$, $t_4 = 11$, $t_5 = 7$, $t_6 = 25$, $b_1 = 7$, $b_2 = 11$, $b_3 = 1$, $b_4 = 11$, $b_4 = 1$, $d_1 = 10$, $d_2 = 10$, $d = 21$, $D = 20$, and $L = 31$.

[1] Kurt Artmann. Calculation of lateral displacement of totally reflected ray. *Ann. Phys.* **437**, 87–102 (1948). URL https://onlinelibrary.wiley.com/doi/10.1002/andp.19484370108.

[2] Beenakker, C. W. J., Sepkhanov, R. A., Akhmerov, A. R. & Tworzydło, J. Quantum Goos-Hänchen effect in graphene. *Phys. Rev. Lett.* **102**, 146804 (2009). URL https://link.aps.org/doi/10.1103/PhysRevLett.102.146804.

[3] Jiang, Q.-D., Jiang, H., Liu, H., Sun, Q.-F. & Xie, X. C. Topological Imbert-Fedorov shift in Weyl semimetals. *Phys. Rev. Lett.* **115**, 156602 (2015). URL https://link.aps.org/doi/10.1103/PhysRevLett.115.156602.

[4] Marchand, D. J. J. & Franz, M. Lattice model for the surface states of a topological insulator with applications to magnetic and exciton instabilities. *Phys. Rev. B* **86**, 155146 (2012). URL https://link.aps.org/doi/10.1103/PhysRevB.86.155146.

[5] Haug, Hartmut and Jauho, Antti-Pekka and others. *Quantum kinetics in transport and optics of semiconductors*, vol. 2 (Springer, 2008).

[6] MacKinnon, A. The calculation of transport properties and density of states of disordered solids. *Z. Physik B - Condensed Matter* **59**, 385–390 (1985). URL https://doi.org/10.1007/BF01328846.

[7] Metalidis, G. & Bruno, P. Green's function technique for studying electron flow in two-dimensional mesoscopic samples. *Phys. Rev. B* **72**, 235304 (2005). URL https://link.aps.org/doi/10.1103/PhysRevB.72.235304.

[8] Jiang, H., Wang, L., Sun, Q.-f. & Xie, X. C. Numerical study of the topological Anderson insulator in HgTe/CdTe quantum wells. *Phys. Rev. B* **80**, 165316 (2009). URL https://link.aps.org/doi/10.1103/PhysRevB.80.165316.

[9] Xiao, D., Chang, M.-C. & Niu, Q. Berry phase effects on electronic properties. *Rev. Mod. Phys.* **82**, 1959–2007 (2010). URL https://link.aps.org/doi/10.1103/RevModPhys.82.1959.

[10] Zhang, Z. *et al.* Non-reciprocal charge transport in an intrinsic magnetic topological insulator $MnBi_2Te_4$. *arXiv:2203.09350 [cond-mat]* (2022). URL http://arxiv.org/abs/2203.09350.
